# Self-reported food allergies in early childhood in rural Australia

**DOI:** 10.3389/falgy.2025.1544496

**Published:** 2025-01-29

**Authors:** Heinrich C. Weber, Gaylene L. Bassett, Sukhwinder S. Sohal, Sarah J. Prior

**Affiliations:** ^1^ Department of Paediatrics, Tasmanian Health Service – Northwest, Burnie, TAS, Australia; ^2^ Tasmanian School of Medicine, Rural Clinical School, University of Tasmania, Burnie, TAS, Australia; ^3^ Respiratory Translational Research Group, Department of Laboratory Medicine, School of Health Sciences, University of Tasmania, Launceston, TAS, Australia; ^4^ Tasmanian School of Medicine, University of Tasmania, Burnie, TAS, Australia

**Keywords:** food allergy, paediatric, childhood, rural, self-reported

## Abstract

**Introduction:**

The prevalence of childhood food allergies is escalating, with Australia notably affected. Research primarily originates from urban centres, leaving rural areas underrepresented. This study examines food allergy prevalence among 1,052 grade 1 and 2 children in regional and rural Tasmania.

**Method:**

Diagnosis relied on validated parental self-reports and identified anaphylaxis by symptoms coupled with breathing difficulties.

**Results:**

The median participant age was 8.1 years. Food allergy prevalence stood at 8.5% (*n* = 89), with cow's milk, peanuts/nuts, and eggs as primary allergens. Anaphylaxis prevalence was 18.0% (*n* = 16) of participants with food allergies, predominantly triggered by peanuts/nuts, eggs, and shellfish.

**Conclusion:**

The study delves into reactions to non-allergenic foods and associated avoidance leading to increased morbidity. This report contributes valuable insights to the insufficiently documented landscape of food allergy prevalence, shedding light on a poorly described aspect.

## 1 Introduction

Allergic diseases are increasingly prevalent worldwide, particularly in developed nations, and Australia stands at the forefront of this trend. Among these, food allergy ranks as one of the most common chronic non-communicable childhood illnesses (1). Termed the “second epidemic” after the rise in asthma and allergic rhinitis, the surge in food allergies, particularly among younger age groups, is a significant contributing factor (2). Unfortunately, food allergies remain inadequately documented on a global scale, with approximately half of all countries lacking prevalence data. Most documented reports on food allergy prevalence primarily stem from developed nations, predominantly major urban centres (1). In Australia, the reported incidence of proven food allergies in children is notably high at 10% (3), compared to the global average of approximately 4% (4). Despite Australia's leadership in food allergy research, much of the existing data originates from major urban areas, leaving rural (regional and remote) areas relatively unexplored. This knowledge gap extends to the overall burden of food allergy, which has lifelong implications for families and society. Its impact, especially in regional and remote areas, remains insufficiently described.

## 2 Methods

We performed a cross-sectional study of all children at 39 schools in Northwest Tasmania, a regional and remote area, and performed a parent-reported questionnaire-based study. All 55 schools in the area were invited to participate by letter and telephonic request addressed to the schools' principals and school boards. Participants were recruited by sending out invitation/information letters to parents.guardians. The questionnaires were completed by parents/guardians during January to November 2014. A completed questionnaires was considered as implied informed consent. Northwest Tasmania is the most remote part of Tasmania and has a lower socio-economic status and higher prevalence of Indigenous population compared to the national average. This area is served by 2 major regional hospitals, smaller rural hospitals and family practices. Acute presentations of food allergies, such as cases of anaphylaxis are generally managed by the paramedic services, as well as the emergency departments at the local hospitals.

Relative geographic remoteness in Australia is objectively measured using the Accessibility/Remoteness Index of Australia Plus (ARIA+) (5). The classes of remoteness are based on relative access to services. The five remoteness classes are: major cities, inner regional, outer regional, remote, and very remote.

Validated questions on food allergies were included in the questionnaire. Validation was based on a reproducible reaction to the same food and symptoms occurring within 1–2 h of ingestion (1, 6). An immediate-type food reaction was defined by answering “yes” to the following questions: (i) a reaction to any food, (ii) always the same food, and (iii) the clinical reaction occurring within 1–2 h of ingestion of the food. Anaphylaxis was identified as an immediate-type reaction to food, with an allergic reaction associated with breathing difficulties.

Stata 12.1 (Statcorp, Texas) software was used for statistical analysis. Results in this prevalence study were recorded as proportions (percentages), with the numerator representing the number of participants with food allergies or anaphylaxis, and the denominator representing the total number of participants. The study was approved jointly by the Tasmanian Human Research Ethics Committee (Reference number H0012975) and the Tasmanian Department of Education. Permission was also obtained from each individual school.

## 3 Results

Of the 1,925 eligible children attending grades 1 or 2 at the 39 participating schools. In terms od remoteness criteria, participating schools were caregorised as located in outer regional areas 33, remote 5 and very remote 1. A total of 1,098 questionnaires were returned. We deemed 1,052 participants eligible for inclusion into the study as a number of participants were excluded from the study due to indecipherable completion or ineligible siblings (i.e., not in grades 1 or 2). We also excluded 10 participants from the study as the questions related to food allergies were not completed adequately. The median age was 8.1 years, a slight male predominance (51.2%), 80% attended public schools whilst 11% were Aboriginal. The prevalence of parent reported food allergies in our study was 8.5% (*n* = 89) and the individual food allergies are outlined in Table 1. Anaphylaxis was reported in 18.0% (*n* = 16) of all participants with food allergies. The food allergens to which they developed anaphylaxis to, are outlined in Table 1. An almost equivalent number of participants (7.9%) who developed food allergies ever, exhibited reactions to foods that they were avoiding in their diet, which are unlikely to be attributed to immediate-type food allergies. These foods include the following: gluten/coeliac 8, fruit & vegetables not known to cause allergies 7, citrus fruit 13, various forms of preservatives 47 and lactose intolerance 11.

**Table 1:** Prevalence of individual food allergens.

Food	Food reactions number (%) (*n* = 1,052)	Anaphylaxis^a^ number (%) (*n* = 1,052)
Cow's milk	25 (2.4%)	2 (0.2%)
Peanut & other nuts	25 (2.4%)	6 (0.6%)
Egg	19 (1.8%)	5 (0.5%)
Strawberries	13 (1.2%)	1 (0.1%)
Wheat	10 (1.0%)	2 (0.2%)
Kiwi fruit	8 (0.8%)	1 (0.1%)
Shellfish	7 (0.7%)	4 (0.4%)
Soy	4 (0.4%)	
Fish	3 (0.3%)	
Banana	3 (0.3%)	
Watermelon	2 (0.2%)	

Individual cases of allergies to honey, ham and coconut.

Total number of participants with food allergies was 89, and those with anaphylaxis was 16.

^a^ Anaphylaxis defined as a reaction to food resulting in breathing problems. In 1 case of anaphylaxis there was uncertainty of the specific food.

## 4 Discussion

Our reported prevalence of parent reported food allergies (8.5%) was similar to previous studies in South Australia (7.3%) and Canada (7.1%) (7, 8). In an earlier groundbreaking Australian study, food allergies in infancy were confirmed at 10% through oral food challenges (3), which is the highest reported prevalence of food allergies in the world. It is generally known that the prevalence of self-reported food allergies is over-reported compared to the gold standard in diagnosis of a food allergy, i.e., oral food challenge. The prevalence in our study compares to the prevalence in urban Australia over a decade earlier (7). Interestingly, it was also considerably lower than the 10% proven allergies in infancy (3). This is even more relevant as it is likely that the true food allergy prevalence is likely lower. Furthermore, in the same study the asthma prevalence was higher than the national average while the food allergy prevalence was lower compared to that reported in urban areas.

The rural urban difference in food allergy prevalence has been demonstrated elsewhere. In another Australian study, proxies for allergy, such as hydrolysed formula and adrenaline, used as a proxy for cow's milk allergies and anaphylaxis presentations, showed an association with higher socio-economic status and urban status (9). In a recent Australian (Victoria) study, there was a higher prevalence of food allergy presentation in paediatric emergency departments (10). In the latter study, over a 10-year period, food allergy related ED presentations increased by 76% in urban ED, compared to a 38% increase in rural areas over the same period. This increased in food allergy related ED presentations predominantly occurred in the 0–4-year age group. In a US study there was also a rural urban difference with odds of 1.7 times more in urban vs. rural areas (11). This difference remained constant once controlled for various other factors, such as race/ethnicity, latitude, gender, age and household income. In another report in Australia and New Zealand a difference in latitude and food allergy prevalence was noted (12). This association with latitude has led to a hypothsised link between UV radiation/sunlight exposure and resulting vitamin D levels and food allergy risk (13). In rural areas, lower food allergies may be influenced by factors like different allergen exposure, proximity to animals, parasitic infestations, UV light, air pollution, probiotic use, microbiome composition, breastfeeding practices, and diet.

The most common food allergens reported in our study were cow's milk, peanut/nuts and egg. These are consistent with international reports where the most common allergens across all regions were cow's milk, egg, peanut and seafood (1). The frequency of these differed across various regions. In our study cow's milk was the most common food allergen, similar to the Canadian study (8). The prevalence of combined peanut and tree nut allergies were however less than the similarly parent-reported study from the Australian Capital Territory a decade earlier (nut ever 3.8% & peanut 3.3%) (14). This was also lower than the proven food allergy prevalence of peanut allergies (2.9%) in urban Victoria a decade earlier (3). Interestingly, about almost 5% of patients reported reactions to preservatives, such reactions also reported in a South Australian study (7). Surprisingly fruit allergens were common such as strawberry and kiwi fruit, with shellfish less commonly reported. It has been reported that up to 60% of such reactions may be related to oral food allergy syndrome, with minimal risk of anaphylaxis (15). Other reported adverse reactions of foods (unlikely to be allergenic) include gluten/coeliac, certain fruits and vegetables not known to cause allergies, citrus fruit, various forms of preservatives and lactose intolerance. Although these latter reactions are unlikely to be attributable to an allergy, the avoidance of such foods still impacts families significantly.

We report peanuts and nuts, egg and shellfish as the most common foods causing food-related anaphylaxis. Although shellfish were not a highly prevalent cause of food allergy, it was responsible for a disproportionately higher (almost 60% of shellfish allergies) prevalence of food-related anaphylaxis in our study. The food allergens causing anaphylaxis in this study were like those reported in studies elsewhere. The prevalence of food-related anaphylaxis was about 1.5%% compared to 0.59% in the South Australian study (7), reported almost 15 years earlier which is most likely in keeping with the time trends of increasing anaphylaxis over time (doubling of anaphylaxis over time). In another study, incorporating the same period, we also report a lower prevalence of childhood anaphylaxis presentations to the ED of local hospitals (16).

A limitation of this study is that the food allergy diagnoses were based on questionnaire-based information, and not on objective oral food challenges. It is widely known that self-reporting of food allergies is most likely over-reporting compared to diagnoses based on oral food challenges (1). Oral food challenges require the necessary expertise, are labour intensive and is not without risk, which limit its use in rural areas. Most of the fruit and vegetable allergies could be attributable to oral allergy syndrome, while about 60% of self-reported cow's milk allergy could be attributable to immediate IgE-mediated allergies (17). Furthermore, the prevalence of anaphylaxis is most likely underreported in this study as the definition of anaphylaxis only incorporated participants with breathing difficulties, and not symptoms related to other symptoms such as gastrointestinal or cardiovascular adverse effects. A mitigating factor is that the vast majority of participants with anaphylaxis in our region presented with respiratory and skin adverse effects (16). Possible sources of bias include selection bias and recall bias. Notably, non-participating schools did not differ in demographic data compared to participating schools. Although non-participation bias is a possibility, several studies have shown that participation rates between 30% and 70% do not substantially affect the ultimate outcomes, suggesting that this effect is often overestimated (18).

## 5 Conclusion

We describe childhood food allergy presentations in a rural environment, generally poorly described in current literature. Although it is likely that fewer patients will have proven food allergies, we describe a lower prevalence of food-related allergies in regional and remote Australia. We also provided an estimate of the burden of patients who are impacted by following unnecessary dietary restrictions and impacted by such an incorrect diagnosis. The responsible food allergens are similar to that described in other rural areas.

## Data Availability

The raw data supporting the conclusions of this article will be made available by the authors, without undue reservation.
